# A novel boronic acid-based fluorescent sensor for selectively recognizing Fe^3+^ ion in real time†

**DOI:** 10.1039/c9ra03978c

**Published:** 2019-06-28

**Authors:** Guiqian Fang, Hao Wang, Zhancun Bian, Min Guo, Zhongyu Wu, Qingqiang Yao

**Affiliations:** School of Medicine and Life Sciences, University of Jinan-Shandong Academy of Medical Sciences Jinan 250200 Shandong China wu_med@foxmail.com yao_imm@163.com; Institute of Materia Medica, Shandong Academy of Medical Sciences Jinan 250062 Shandong China; Key Laboratory for Biotech-Drugs Ministry of Health Jinan 250062 Shandong China; Key Laboratory for Rare & Uncommon Diseases of Shandong Province Jinan 250062 Shandong China; Shandong Leather Industrial Research Institute Jinan 250021 Shandong China

## Abstract

Boronic acid provides faster fluorescence response to Fe^3+^ compared to other reported sensors, which is critical for continuous dynamic detection. Herein, we reported a novel boronic acid-based sensor 4 that could recognize Fe^3+^ ion in real time. After 10 equiv. of Fe^3+^ ion (1 mM) was added, the fluorescence of sensor 4 was immediately quenched by 96%. While other ions, including Ba^2+^, Ca^2+^, Cr^2+^, Cd^2+^, Co^2+^, Cs^2+^, Cu^2+^, Fe^2+^, K^+^, Li^+^, Mg^2+^, Mn^2+^, Na^+^, Ni^2+^ or Zn^2+^, respectively, did not change the fluorescence significantly. Further tests indicated that the high selectively sensing Fe^3+^ ion benefits from the two boronic acid functionalities in the structure. Moreover, interference experiments showed this sensor has an excellent anti-interference ability. In addition, we performed binding activity test in rabbit plasma and other real samples for practical applications, obtaining similar results. And the thin layer loading sensor 4 was also successfully applied to recognize Fe^3+^ ion among various ions. Therefore, 4 may serve as a potential sensor for continuous monitoring and detecting Fe^3+^ ion in real time.

## Abstract 2

Boronic acid provides faster fluorescence response to Fe^3+^ compared to other reported sensors, which is critical for continuous dynamic detection.

## Graphical abstract

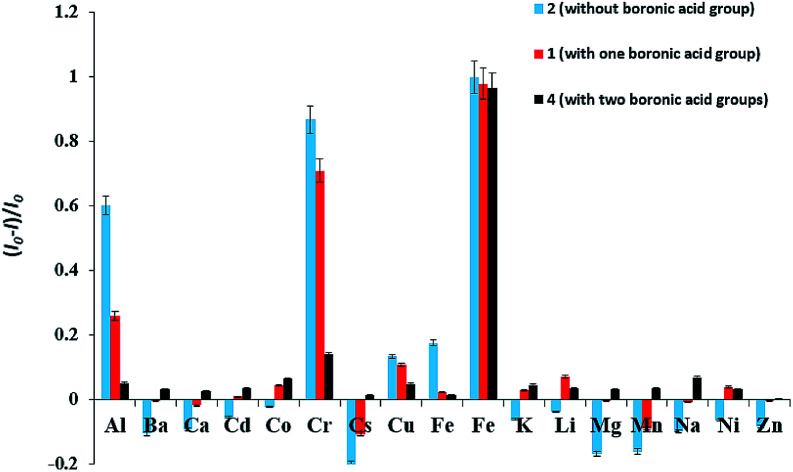

## Introduction

Among various trace elements, Fe^3+^ ion is one of the most abundant essential elements in human physiological activities. As an important component for the formation of hemoglobin, myoglobin and various enzymes, Fe^3+^ ion participates in many biological and chemical processes at the cellular level and plays an important role in transporting oxygen and nutrients in the blood.^1–3^ A lack or excess of Fe^3+^ ion may lead to low immunity, reduced intelligence and reduced anti-infective capacity, affecting the body's ability to regulate body temperature, and even inducing a variety of diseases, such as Alzheimer's disease, Parkinson's syndrome and other neurodegenerative diseases.^4–6^ In daily life, Fe^3+^ ion is added to many foods, health care products, fertilizers and pesticides to promote its absorption by humans and crops. Studies have shown that many diseases, such as heart disease, diabetes mellitus and even hepatocellular cancer, are associated with excessive Fe^3+^ ions intake in living organisms.^4^ Therefore, to establish a high selectivity and high sensitivity Fe^3+^ ion detection method has an important theoretical and practical significance. Currently, the detection method of Fe^3+^ ion depends on voltammetry,^7^ atomic absorption spectrometry,^8^ fluorescence spectroscopy^6^ and so on. However, the selectivity of voltammetry is poor, and atomic absorption spectroscopy needs higher requirements on instruments and more expensive analysis cost. Due to the simple operation, high sensitivity and selectivity, fluorescence spectroscopy has been widely applied to detect various ions (Cu^2+^,^9–15^ Cr^3+^,^16^ Hg^2+^,^17–21^ Al^3+^,^22,23^*etc.*), carbohydrates^24–27^ and so on. Compared with sensing other transition metals such as Cu^2+^ and Hg^2+^, there are relatively less fluorescent sensors of Fe^3+^ ion, which are based on derivatives of macrocyclic molecules,^2,28^ rhodamines,^29–34^ coumarin,^35–37^ quinoline,^1,38–40^*etc.* However, most Fe^3+^ ion sensors reported have irreversible recognition,^30,41^ long response time,^1,29,36,42^ poor water solubility,^28,42,43^ and are susceptible to interference from other transition metal ions such as Zn^2+^, Cr^3+^ and Pb^2+^.^31,44,45^ Therefore, the development of highly selective and sensitive Fe^3+^ ion sensors still remains a challenge.

Due to the obvious changes in fluorescence after binding, rapid recognition, good selectivity, *etc.* boronic acid-based fluorescent sensors have been developed widely in the recognition of carbohydrates,^24–27^*etc.* In addition, boronic acid can also be used to sense ions,^11,17^ such as Cu^2+^, Hg^2+^ and so on. However, the selective recognition of Fe^3+^ ion by boronic acid-based sensors has not been reported yet. It is necessary to emphasize the role of boronic acid groups in the recognition of unreported metal ions including Fe^3+^ ion. 2-(4-Boronophenyl)quinoline-4-carboxylic acid (PBAQA/sensor 1) is a water-soluble fluorescent sensor reported by Wang *et al.*^46^ Our group is dedicated to the synthesis and applications of PBAQA and its derivatives.^47^ In this paper, a diboronic acid sensor 4 was synthesized, which selectively recognizes Fe^3+^ ion.

**Scheme 1:**
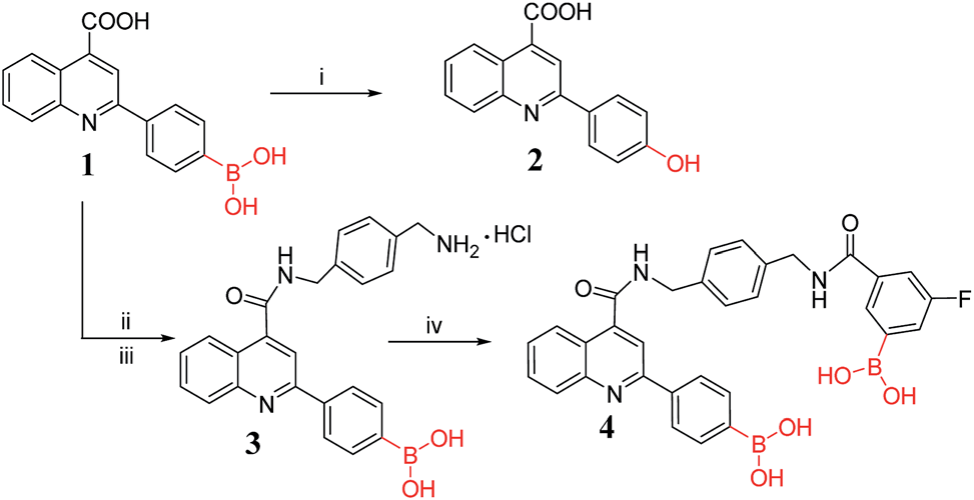
Synthetic route of target compounds. (i) 30% H_2_O_2_, rt, 4 h. (ii) 1-(*N*-Boc-aminomethyl)-4-(aminomethyl)benzene, 4-(4,6-dimethoxy-1,3,5-triazin-2-yl)-4-methylmorpholinium, rt, 16 h. (iii) HCl, rt, 4 h. (iv) 3-Carboxy-5-fluorobenzeneboronic acid, 4-(4,6-dimethoxy-1,3,5-triazin-2-yl)-4-methylmorpholinium, triethylamine, rt, 16 h.

Sensor 1 was used as the building block for the synthesis of diboronic compounds. And the synthesis method of sensor 1 has been reported by our group.^47^ In the acylation reaction, using DMT-MM as the condensing agent to activate the carboxyl group could achieve more desirable results. DMT-MM was used in the two-step amide condensation reaction carried out at room temperature in Scheme 1, completely avoiding the use of environment-hazardous SOCl_2_. Methanol acted as solvent instead of DMF, which was easily removed by concentration under reduced pressure after the reaction was completed. Therefore, the synthesis method was more environmentally friendly than the previous synthesis method.^47,48^ The structures of intermediate compound 3 and the target compound 4 were confirmed by HRMS and NMR. Subsequently, preliminary fluorescence activity screening was performed, and we found that compound 4 has certain selectivity for Fe^3+^ ion.

## Experimental section

### Materials and physical measurements

All materials for synthesis were commercially available without further purification. All solvents used were of analytical reagent grade. All aqueous solutions were prepared using pure water. The solutions of the metal ions were prepared from their chloride salts and the solutions of the anions were prepared from their sodium salts.

Fluorescence spectra were measured using RF5301PC Fluorescence Spectrophotometer (Shimadzu, Japan). The NMR spectra were recorded on with a Bruker AM-600 spectrometer (Billerica, MA) with tetramethylsilane as the internal standard. High resolution mass spectra (HRMS) were recorded on an Agilent 1290LC-6540 Accurate Mass Q-TOF by using electrospray ionization (ESI).

### Syntheses and characterization

The hydroxyl derivative, compound 2 was prepared according to the established in literature.^49^

#### Synthesis of compound 2

A 30% H_2_O_2_ (3 mL) solution was added drop-wise to a solution of compound 1 (0.15 g, 5.1 × 10^−4^ mol) in methanol (20 mL), then stirred for 4 h at room temperature. A yellow solid precipitated and sensor 1 was shown to be completely reacted by thin layer chromatography (TLC). The solvent was removed by filtration and brown solid was obtained. The crude product was washed with cold ethyl ester and ethanol and dried to get a yellow powder compound 2 (100 mg, 74%). The color was the same as described in the literature.^49^ HRMS *m*/*z* (Fig. S1†): calculated 266.0812, found 266.0797.

#### Synthesis of compound 3

A solution of compound 1 (0.2 g, 6.8 × 10^−4^ mol), 1-(*N*-Boc-aminomethyl)-4-(aminomethyl)benzene (0.140 g, 7.5 × 10^−4^ mol) and 4-(4,6-dimethoxy-1,3,5-triazin-2-yl)-4-methylmorpholinium (0.200 g, 7.5 × 10^−4^ mol) in methanol (20 mL) was added a drop of *N*-methylmorpholine in a bottom flask. The mixed solution was protected with nitrogen atmosphere, and stirred at room temperature for 16 h (25 °C). After the reaction was completed, the reaction mixture was added to ice water and stirred. A yellow powder solid was obtained by filtration, and was washed three times by water and then recrystallized from methanol–water. After vacuum drying, a light yellow powder product was obtained. A solution of the light yellow powder product in ethyl acetate (30 mL) was added 1 mL of hydrochloric acid slowly and stirred for 4 h at room temperature. The reaction mixture changed from a yellow clarified state to a yellow turbid state. After vacuum drying, a light yellow powder compound 3 was obtained (0.202 g, 66%). ^1^H NMR (600 MHz, DMSO-*d*_6_) *δ* (ppm) (Fig. S2†): 9.64 (t, *J* = 5.7 Hz, 1H), 8.54 (s, 3H), 8.32–8.21 (m, 5H), 8.02 (d, *J* = 7.9 Hz, 2H), 7.89 (t, *J* = 7.5 Hz, 1H), 7.70 (t, *J* = 7.6 Hz, 1H), 7.50 (dd, *J* = 22.3, 8.0 Hz, 4H), 4.62 (d, *J* = 5.8 Hz, 2H), 4.01 (d, *J* = 5.6 Hz, 2H). ^13^C NMR (151 MHz, DMSO-*d*_6_) *δ* (ppm) (Fig. S3†): 166.84, 156.17, 147.11, 144.16, 139.78, 138.84, 136.88, 135.15, 133.22, 131.34, 129.60, 128.89, 128.14, 127.99, 127.14, 125.89, 124.08, 117.94, 42.92, 42.37. HRMS *m*/*z* (Fig. S4†): calculated 412.1827, found 412.1807.

#### Synthesis of compound 4

A solution of compound 3 (0.11 g, 2.5 × 10^−4^ mol), triethylamine (0.042 mL), 3-carboxy-5-fluorobenzeneboronic acid (0.046 g, 2.75 × 10^−4^ mol) and 4-(4,6-dimethoxy-1,3,5-triazin-2-yl)-4-methylmorpholinium (0.076 g, 2.75 × 10^−4^ mol) in methanol (15 mL) was added a drop of *N*-methylmorpholine. The bottom flask was protected with nitrogen atmosphere, stirred at room temperature for 16 h at room temperature. After the reaction was completed, the reaction mixture was added to ice water and stirred. A yellow powder solid was obtained by filtration, which was washed three times with water and recrystallized from methanol–water. After vacuum drying, a light yellow powder compound 4 was obtained (0.092 g, 64%). ^1^H NMR (600 MHz, MeOD) *δ* (ppm) (Fig. S5†): 8.20–8.10 (m, 4H), 8.04 (s, 1H), 7.82 (dd, *J* = 52.3, 44.8 Hz, 4H), 7.60–7.44 (m, 4H), 7.40–7.30 (m, 3H), 4.70 (s, 2H), 4.62 (s, 2H). ^13^C NMR (151 MHz, MeOD) *δ* (ppm) (Fig. S6†): 168.50, 148.30, 143.14, 139.32, 138.71, 133.74, 130.08, 129.01, 128.54, 127.11, 126.41, 126.35, 126.32, 126.30, 124.84, 123.51, 123.49, 116.83, 43.18. HRMS *m*/*z* (Fig. S7†): calculated 578.2065, found 578.2060.

## Results and discussion

### Photophysical analysis

A sensor stock solution (10^−3^ M) was prepared in DMSO and the ion stock solution (10^−2^ M) was prepared in pure water. And 1 mL of the sensor (1 × 10^−4^ M) was prepared with different ion concentration (0 to 10^−3^ M). UV-vis absorption spectra of sensor 1, 2 and 4 was recorded in DMSO/H_2_O (3 : 7, v/v). It can be seen from Fig. S8† that the maximum UV-vis absorption wavelength of sensor 1, 4 are at around 337 nm and sensor 2 is at around 340 nm. The excitation wavelength of sensor 1 and 4 were set at 337 nm (slit: 5 nm/5 nm). The excitation wavelength of sensor 2 was set at 340 nm (slit: 5 nm/5 nm).

**Fig. 1:**
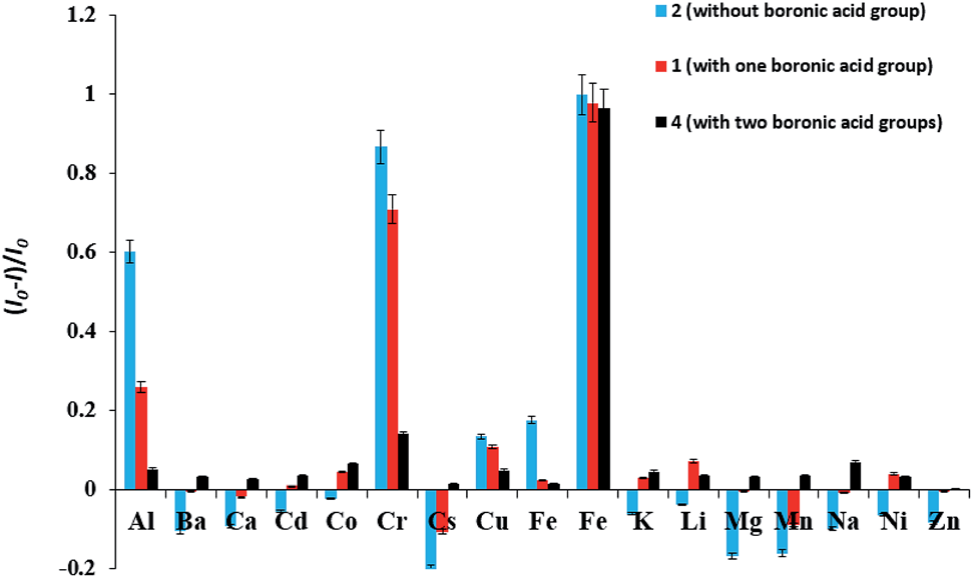
Relative fluorescence intensity of sensor 1, 2, 4 (1 × 10^−4^ M) in the absence and presence of 10 equiv. of various metal ions in DMSO/H_2_O (3 : 7, v/v) solution, at room temperature.

We used sensor 1, 2 and 4 to screen the preliminary fluorescence activity of metal ions. These three compounds have significant fluorescent response to Fe^3+^ ion. Among them, none boronic acid-based sensor 2 also has a large fluorescent response to Al^3+^, Cr^2+^, followed by the monoboronic acid sensor 1, while diboronic acid sensor 4 is not significant fluorescent response for the two metal ions, showing a higher selectivity for Fe^3+^ ion, as shown in Fig. 1. This indicates that the boronic acid group plays an important role in increasing the selectivity to Fe^3+^ ion.

### The response time of sensor 4

**Fig. 2:**
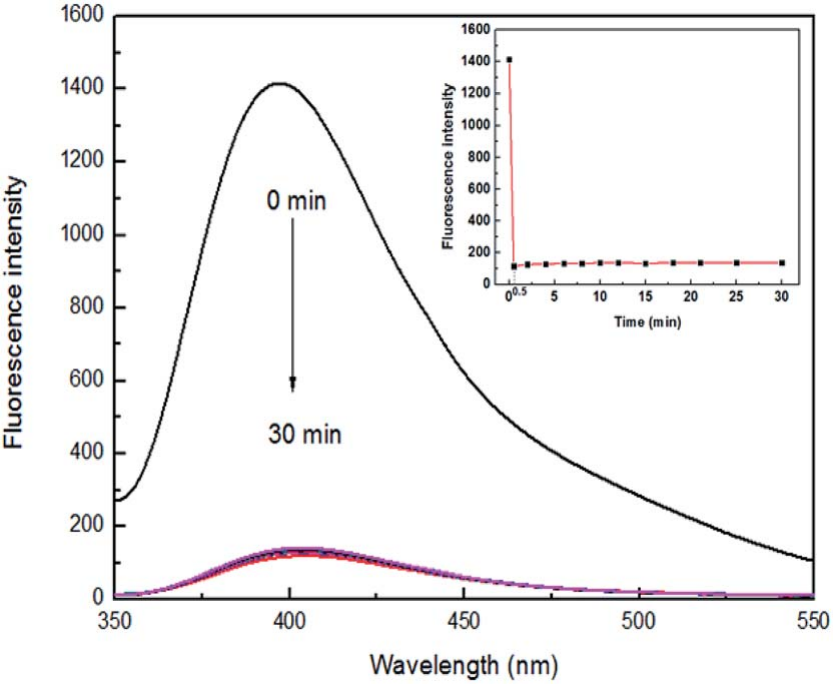
Fluorescence spectra of sensor 4 (1 × 10^−4^ M) upon addition of 7 equiv. of Fe^3+^ ion from 0 to 30 min in DMSO/H_2_O (3 : 7, v/v), at room temperature. Inset: plot of the fluorescence intensities at 400 nm over a period of 30 min.

High sensitivity is a very important aspect for a chemical sensor used to practical applications. To evaluate the sensitivity of sensor 4 toward Fe^3+^ ion, the response time experiment of the reaction system was necessary to carry out. A mixture of sensor 4 (1 × 10^−4^ M) and 7 equiv. of Fe^3+^ ion in DMSO/H_2_O (3 : 7, v/v) was prepared. During one time required to record a change in fluorescence (0.5 min), Fe^3+^ ion was able to quench the fluorescence of 4 in real time and reached saturation, as shown in Fig. 2. There are previously reported that Fe^3+^ ion fluorescent sensors need a long response time. For example, a fluorescent sensor for Fe^3+^ ion reported by Gao and his colleagues requires at least 60 min for mixing of Fe^3+^ ion and sensor before recording a change in fluorescence.^42^ However, sensor 4 can perform a fluorescence test within a time (0.5 min) at which the fluorescence change is recorded. Furthermore, after fluorescence quenching, the fluorescence intensity does not change up and down with the extension of the mixing time, exhibiting excellent stability. Therefore, 4 may serve as a potential sensor for continuous monitoring and detecting Fe^3+^ ion in real time.

### Fluorescent response

**Fig. 3:**
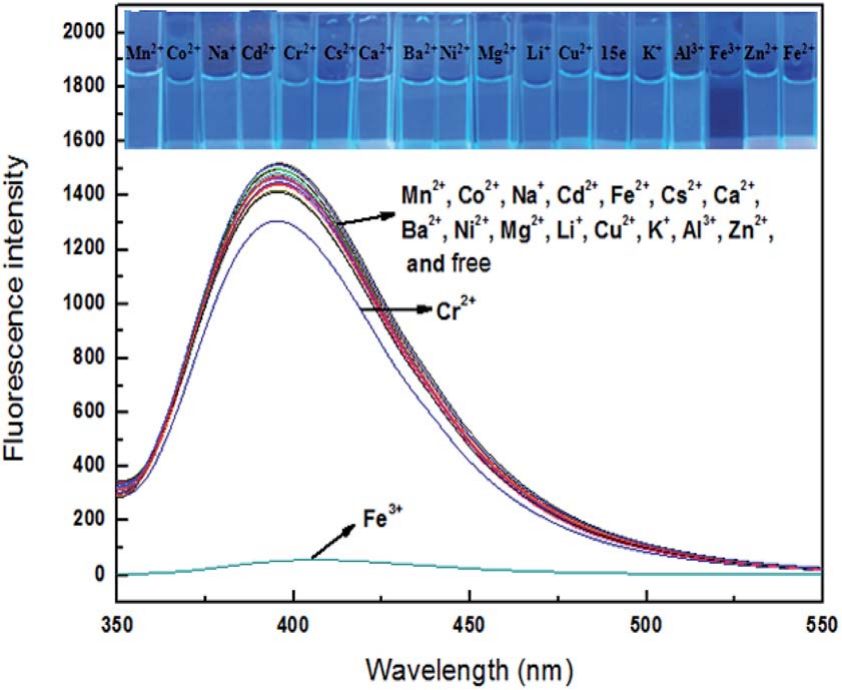
Fluorescence spectra of sensor 4 (1 × 10^−4^ M) in the absence and presence of 10 equiv. of various metal ions in DMSO/H_2_O (3 : 7, v/v): Ba^2+^, Ca^2+^, Cr^2+^, Cd^2+^, Co^2+^, Cs^2+^, Cu^2+^, Fe^2+^, Fe^3+^, K^+^, Li^+^, Mg^2+^, Mn^2+^, Na^+^, Ni^2+^ and Zn^2+^. Inset: photograph of 4 (1 × 10^−4^ M) in presence of 10 equiv. of various ions in DMSO/H_2_O (3 : 7, v/v), which was observed under a UV-lamp (365 nm) (*λ*_ex_ = 337 nm; *λ*_em_ = 400 nm).

Sensor 4 (1 × 10^−4^ M) emitted a steady light blue fluorescence under a UV-lamp (365 nm). Interesting, after adding 10 equiv. of Fe^3+^ ion, the blue fluorescence immediately turned into none fluorescence. However, after adding the same equivalents of Ba^2+^, Ca^2+^, Cr^2+^, Cd^2+^, Co^2+^, Cs^2+^, Cu^2+^, Fe^2+^, K^+^, Li^+^, Mg^2+^, Mn^2+^, Na^+^, Ni^2+^ and Zn^2+^, the solutions still kept blue fluorescence under a UV-lamp (365 nm). Subsequently, we performed fluorescence recording on mixture of these sensor–ion solution and found that the fluorescence intensity of sensor 4 was immediately quenched by 96% after the addition of Fe^3+^ ion, while the fluorescence intensity of sensor 4 with other ions was not significantly changed, respectively, as shown in Fig. 3. Therefore, sensor 4 may be used to selectively recognize Fe^3+^ ion by fluorescence.

**Fig. 4:**
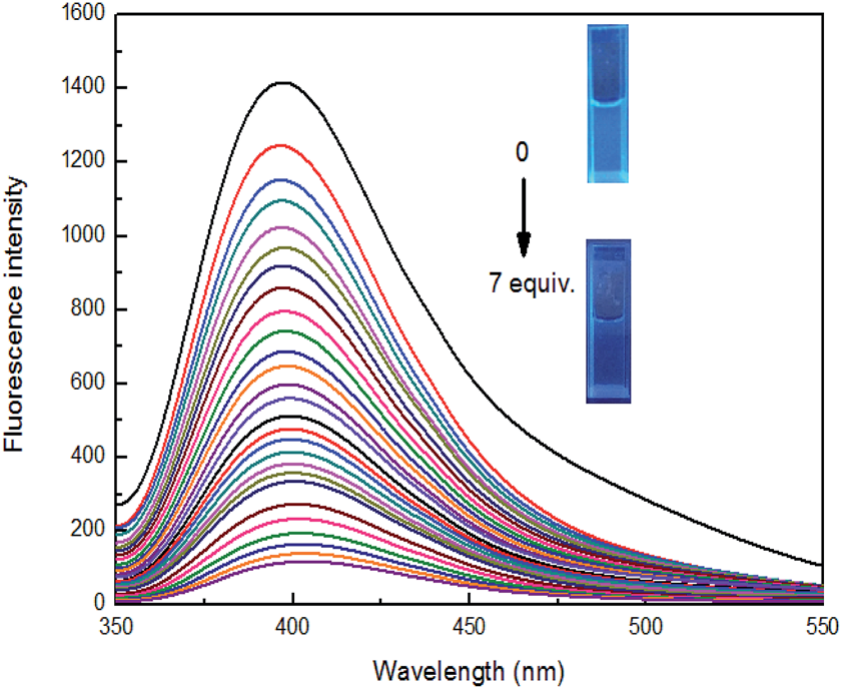
Fluorescence spectra of 4 (1 × 10^−4^ M) in the presence of different concentrations of Fe^3+^ ion in DMSO/H_2_O (3 : 7, v/v), at room temperature. Inset: photograph of 4 (1 × 10^−4^ M) in presence of Fe^3+^ ion in DMSO/H_2_O (3 : 7, v/v), which was observed under a UV-lamp (365 nm) (*λ*_ex_ = 337 nm; *λ*_em_ = 400 nm).

**Fig. 5:**
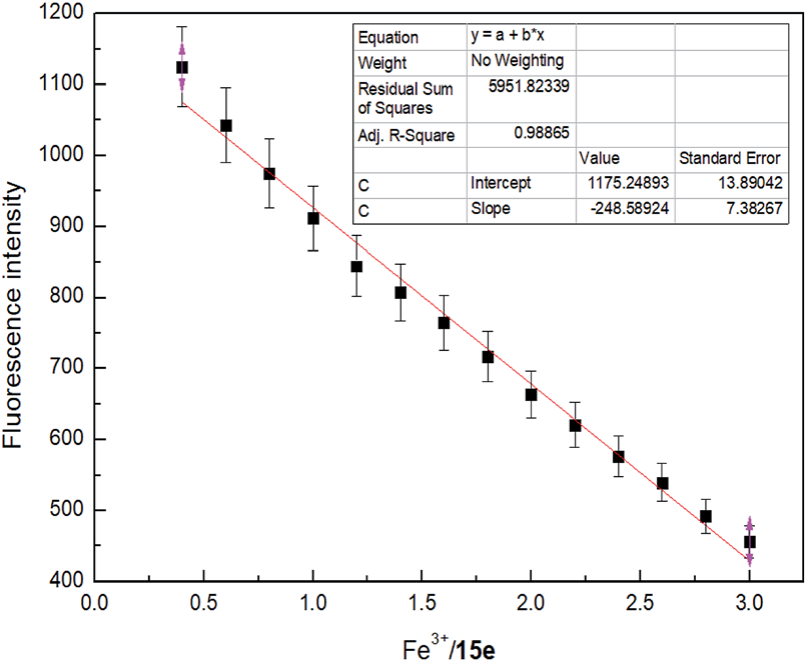
Linear relationship between sensor 4 and Fe^3+^ ion in DMSO/H_2_O (3 : 7, v/v).

To further investigate the significant quenching of the fluorescence of sensor 4 by Fe^3+^ ion, we performed Fe^3+^ ion fluorescence titration test on sensor 4, as shown in Fig. 4. Different equivalents of Fe^3+^ ion was added to sensor 4 (1 × 10^−4^ M) solution and the fluorescence spectra was recorded. As the concentration of Fe^3+^ ion gradually increased, the fluorescence of sensor 4 gradually decreased to almost complete quenching, and the color of solution changed from blue to colorless under a UV-lamp (365 nm). Moreover, it was found a good linear relationship between fluorescence intensity of sensor 4 and the concentration of Fe^3+^ ions in the range of from 4 × 10^−5^ to 30 × 10^−5^ M with correlation coefficient of *R*^2^ = 0.98865, as shown in Fig. 5. The limit of detection (LOD) was then calculated to 6.79 × 10^−7^ M with the following equation:^50–52^LOD = 3*δ*/*S*where *δ* is the standard deviation of the 8 times blank signal of sensor, and *S* is the slope of the calibration curve.

The calculation process can be found in Fig. S9.† In addition, it is worth mentioning that the LOD (6.0 × 10^−6^ M) is close to the US EPA maximum allowable limit for Fe^3+^ ion (0.3 mg L^−1^) in drinking water (Fig. S9†).^1^

### Interference experiments

**Fig. 6:**
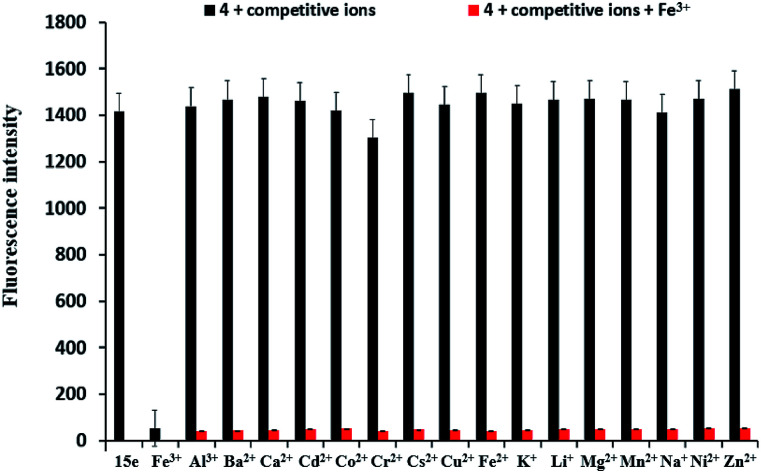
Fluorescence intensities of 4 (1 × 10^−4^ M) in the presence of various metal ions in DMSO/H_2_O (3 : 7, v/v) solution, at room temperature. Black bars represent the fluorescence intensity (400 nm) of 4 in the presence of 10 equiv. of various metal ions (Ba^2+^, Ca^2+^, Cr^2+^, Cd^2+^, Co^2+^, Cs^2+^, Cu^2+^, Fe^2+^, Fe^3+^, K^+^, Li^+^, Mg^2+^, Mn^2+^, Na^+^, Ni^2+^ and Zn^2+^). Red bars represent the fluorescence intensity (400 nm) in the presence of various metal ions after the addition of Fe^3+^ ion.

To further evaluate the selectivity of sensor 4 for Fe^3+^ ion, the interference experiments of sensor 4 (1 × 10^−4^ M) for Fe^3+^ ion was carried out in the presence of 10 equiv. of Ba^2+^, Ca^2+^, Cr^2+^, Cd^2+^, Co^2+^, Cs^2+^, Cu^2+^, Fe^2+^, K^+^, Li^+^, Mg^2+^, Mn^2+^, Na^+^, Ni^2+^ and Zn^2+^ under the same conditions, as shown in Fig. 6. When other metal ions were added to the sensor 4–Fe^3+^ mixed solution, the fluorescence intensities of the metal ions were no significant variation, indicating that sensor 4 has an excellent anti-interference ability and may be used as a selective Fe^3+^ ion fluorescent sensor.

### Reversibility study

**Fig. 7:**
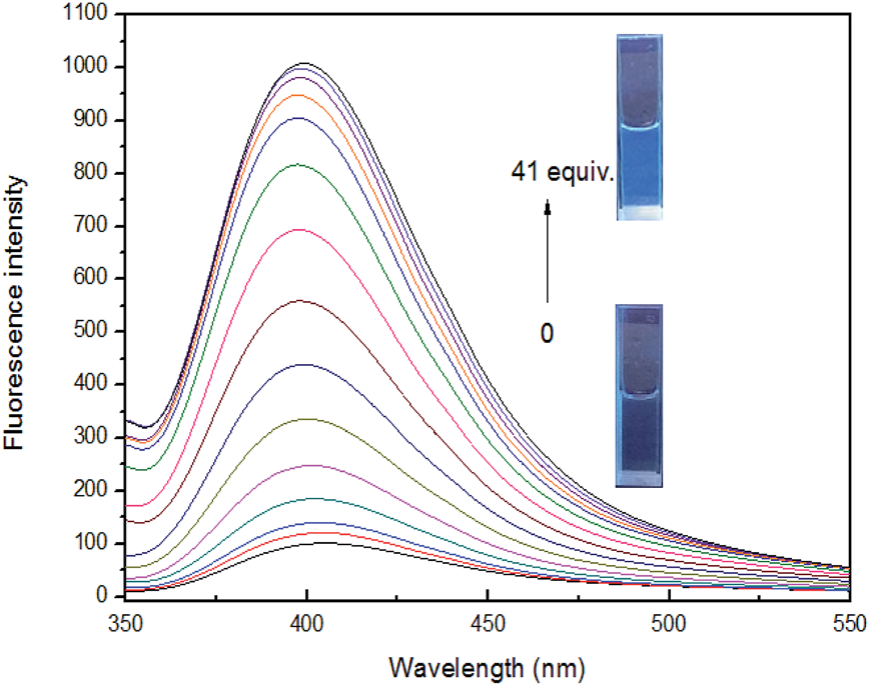
Fluorescence spectra of 4 (1 × 10^−4^ M) with 7 equiv. of Fe^3+^ ion in the presence of different concentrations of F^−^ in DMSO/H_2_O (3 : 7, v/v), at room temperature. Inset: photograph of 4 (1 × 10^−4^ M) with 7 equiv. of Fe^3+^ ion upon adding 41 equiv. of F^−^, which was observed under a UV-lamp (365 nm).

The above studies have shown that sensor 4 can selectively recognize Fe^3+^ ion by fluorescence, and its 4–Fe^3+^ complex exhibits almost complete fluorescence quenching. Owing to F^−^ is known to have a strong binding ability to Fe^3+^ ion,^53^ we wonder if we can introduce F^−^ into investigate the reversibility of 4–Fe^3+^ complex. As the concentration of F^−^ increases to 41 equiv., the fluorescence intensity tends to be stable and no longer increases. The solution is converted from non-fluorescent to faint blue fluorescence, but the emitted fluorescence is weaker than the fluorescence of the blank sensor 4 under a UV-lamp (365 nm). This may be ascribed that Fe^3+^ ion from 4–Fe^3+^ complex is not completely captured by F^−^ ion, and the complex formed by F^−^ and Fe^3+^ ion increases the possibility of interference in the system, so that the fluorescence intensity is slightly weaker than that of blank sensor 4, indicating that binding process is reversible, as shown in Fig. 7.

### Solvent polarity ratio and pH range studies

**Fig. 8:**
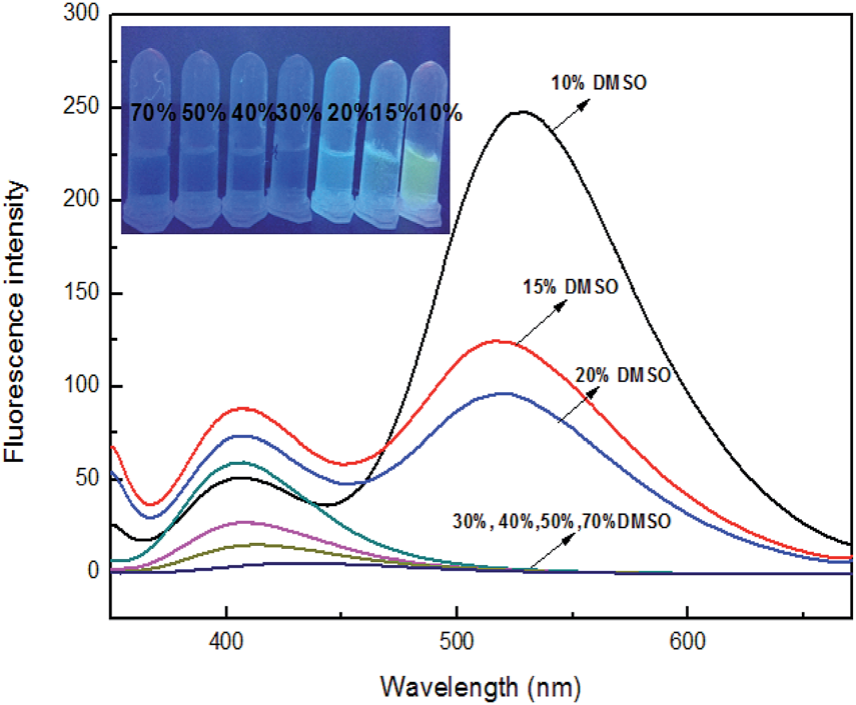
Fluorescence spectra of sensor 4 (10^−4^ M) upon addition of Fe^3+^ (10^−3^ M) in different ratio of DMSO–H_2_O. Inset: photograph of 4 (10^−4^ M) upon adding 10 equiv. of Fe^3+^ in different ratio of DMSO, which was observed under a UV-lamp (365 nm).

To investigate the solvent effects, different polar solvents were carried out for fluorescence testing, including *N*,*N*-dimethylformamide, dimethylsulfoxide, acetone, methanol, ethanol, acetonitrile. However, it was found no significant difference in all solvents (Fig. S10†). Subsequently, a range of different ratios of organic solvent were prepared for determining appropriate solvent ratio for fluorescence testing. Sensor 4 has the maximum fluorescence emission intensity at 400 nm. However, upon adding 10 equiv. of Fe^3+^ ion, the fluorescence of 4 was decreased at 400 nm and had fluorescence enhanced at 529 nm in DMSO/H_2_O (1 : 9, v/v). Interesting, as the proportion of DMSO increased, the fluorescence emitted from sensor 4–Fe^3+^ ion system was observed decreasing gradually under UV-lamp (365 nm), as shown in Fig. 8. In the ratio of 10% DMSO, this phenomenon also occurred in most other ions, as shown in Fig. S10.† Fluorescence was observed from the insoluble precipitates from the UV-lamp (365 nm), which may be due to the addition of ions leading to the precipitation of sensor–Fe^3+^ complex. And the precipitation would bother fluorescence tests. When the ratio reaches 30%, there is no longer any yellow fluorescence releasing, which may be that 30% DMSO is enough for sensor–Fe^3+^ complex to dissolve. Therefore, 30% DMSO is a suitable ratio for fluorescence activity studies. In addition, it is necessary to investigated suitable pH range for the interesting process of sensor 4 recognizing Fe^3+^ ion. As can be seen from the Fig. S11,† under the same condition, sensor 4 has a large fluorescence response in the range of pH 2 to 10.

### The practical applications of sensor 4

**Fig. 9:**
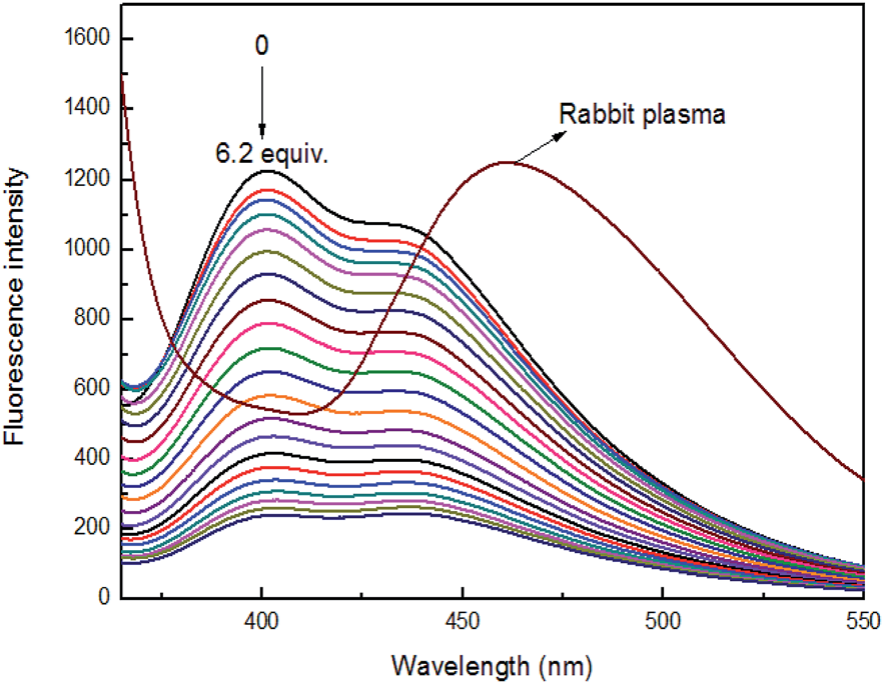
Fluorescence spectra of 4 (1 × 10^−4^ M) in the presence of different concentrations of Fe^3+^ ion in rabbit plasma (3 : 7, v/v), at room temperature.

To investigate the practical applications of sensor 4 for Fe^3+^ ion detection, rabbit plasma was used as biological fluids instead of buffer solution for detecting Fe^3+^ ion. Under the same conditions, the maximum fluorescence emission peak of rabbit plasma appeared at 460 nm, which had no effect on the maximum fluorescence emission peak (400 nm) of sensor 4. Moreover, the prepared sensor 4 rabbit plasma solution has a quenching effect on the fluorescence peak of the rabbit plasma itself, as shown in Fig. 9. It was found a good linear relationship between fluorescence intensity of sensor 4 and the concentration of Fe^3+^ ion in the range of from 2.5 × 10^−5^ to 30 × 10^−5^ M with correlation coefficient of *R*^2^ = 0.98478, as shown in Fig. S13.†

**Fig. 10:**
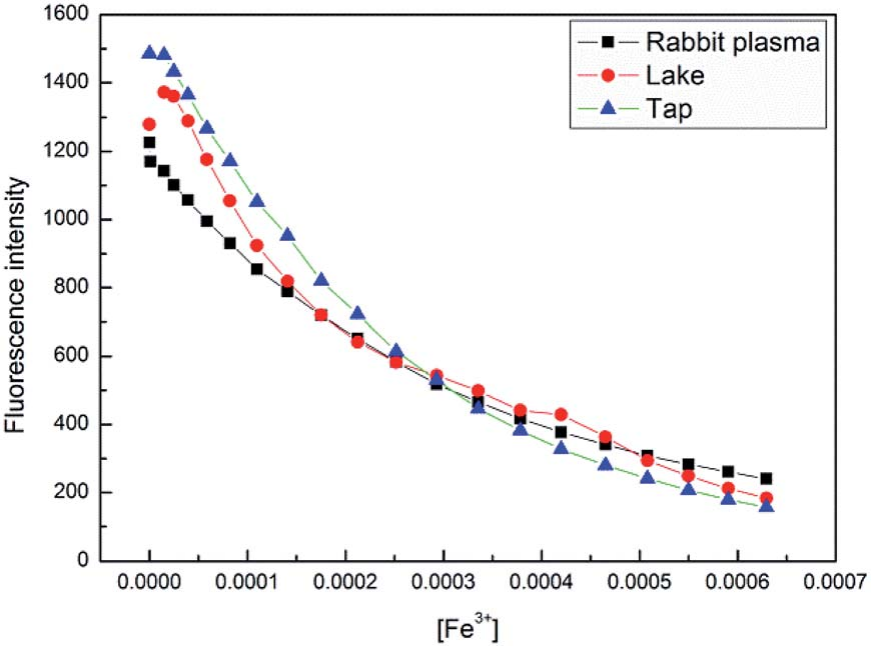
Fluorescence intensity change of 4 (1 × 10^−4^ M) at 400 nm upon continuous addition of Fe^3+^ ion (0 to 60 × 10^−5^ M) in three natural water samples, at room temperature.

The potential practical applications of sensor 4 in sensing the Fe^3+^ ion was also tested in real samples as well as biological samples. The lake water was collected from the lakes of Quan Cheng Gong Yuan, and the tap water was collected from the laboratory. The results of the fluorescence data were observed from these experiments, as shown in Fig. 10. It was found that the fluorescence intensities were proportional to the concentrations of Fe^3+^ ion in the range of from 5 × 10^−5^ to 60 × 10^−5^ M. This study indicates that sensor 4 has a good anti-interference ability and can successfully detect Fe^3+^ ion in real samples.

**Fig. 11:**
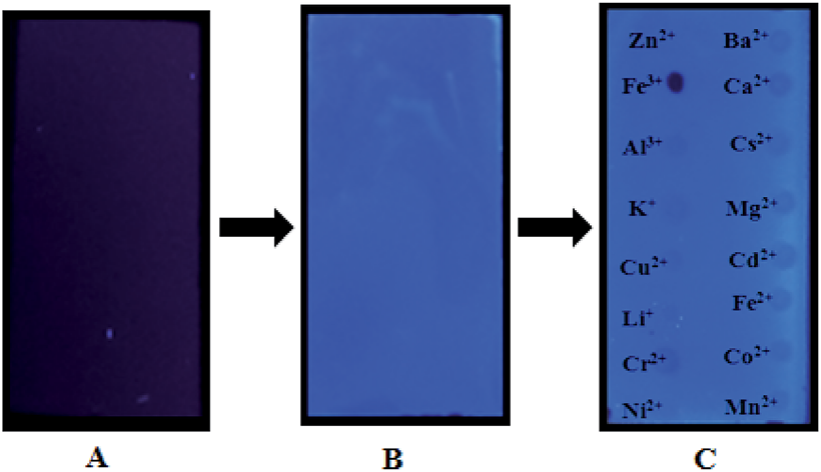
(A) is an untreated thin layer plate, (B) is a thin layer plate treated by 4 (1 × 10^−4^ M), and (C) is a 4-treated thin layer plate with spots sample of various metal ions (1 × 10^−3^ M). All photographs were observed under a UV-lamp (365 nm).

In addition, we used thin layer as a solid phase carrier to assist 4 in the detection of Fe^3+^ ion. The metal ions of aqueous solutions were spotted on a thin layer treated by 4 stock solution (1 × 10^−4^ M), respectively, followed by dying in the air for about 5 min. Interestingly, it was found that Fe^3+^ ion almost completely quenched the fluorescence of 4-treated thin layer plate, while other metal ions were no significant variation under a UV-lamp (365 nm), indicating that 4 may be used as a selective fluorescent sensor for detecting Fe^3+^ ion, as shown in Fig. 11. To the best of knowledge, there is a fewer example of solid films used to sensing Fe^3+^ ion in aqueous solution. Thus, 4 holds a potential to develop into a convenient tool for detecting Fe^3+^ ion.

To gain binding ratio, HRMS analysis of sensor 4–Fe^3+^ complex was further studied. It was found that the molecular weight was equal to that of sensor 4–2Fe^3+^ (calculated 689.0658, found 689.3108), as shown in Fig. S14.† Therefore, the binding ratio of sensor 4 to Fe^3+^ is determined to 1 : 2.^54,55^

The boronic acid groups can form a complex with the metal ions, and the selectivity to the metal ions was reported to be improved by increasing the boronic acid groups, including Swamy *et al.* designed diboronic acid sensor for Cu^2+^, Kim *et al.* reported sensors for Hg^2+^ and so on.^11,17^ However, to the best of our knowledge, there is no boronic acid-based sensor reported for Fe^3+^ ion and we reported the first one. Over past years, there are a good number of fluorescent sensors for Fe^3+^ have been reported, which are usually based on derivatives of macrocyclic molecules,^2,28^ rhodamines,^29–34^ coumarin,^36,37,56,57^ quinoline,^1,38–40^*etc.* These mother core structures usually were further modified with other functional groups, such as Schiff base.^1,36,56^ However, boronic acid groups may be offer a new potential choice. Due to boronic acid groups can form a complex with the metal ions quickly, boronic acid-based sensors could be applied to sense metal ions in a real time. Furthermore, the selectivity of diboronic acid-based sensor 4 to Fe^3+^ was found to be improved when compared to monoboronic acid sensor 1 and none boronic acid-based sensor 2. It is interesting that none boronic acid-based sensor 2 has a large fluorescent response to Al^3+^, Cr^2+^, followed by the monoboronic acid sensor 1, while diboronic acid sensor 4 is not significant fluorescent response for the two metal ions, showing a higher selectivity for Fe^3+^ ion. This may be ascribed to the role of boronic acid group in regulating the process of recognizing Fe^3+^ ion. As far as we know, the sensors for Fe^3+^ ion reported previously mostly enhance the binding ability of binding sites such as Schiff base to Fe^3+^ ion for improving selectivity, while few reports are increasing the selectivity to Fe^3+^ ion by reducing the affinity to other metal ions. As for how the boronic acid group regulates in the process of recognizing Fe^3+^ to reduce the affinity to other metal ions, we need to carry out more experiments to conduct research.

Among the previously reported fluorescence sensors for Fe^3+^ ion, the proportion of organic solvents is quite large. Wei *et al.* reported the fluorescence sensor for Fe^3+^ containing 80% DMSO,^51^ and even 100% DMSO is used for fluorescence activity studies.^42^ The use of a large proportion of organic solvents for fluorescence testing reflects the poor water solubility of sensors, which is not conducive to later practical applications, especially cell experiments (high proportions of DMSO are toxic to cells). Sensor 4 has a better water solubility than those previously reported, and is suitable to detect Fe^3+^ ion in biological fluid, such as rabbit plasma. Currently, Fe^3+^ sensors reported have not met the research needs. The fluorescent sensor for Fe^3+^ ion reported by Gao and his colleagues has a high affinity to Fe^3+^ ion, but the response time is more than 60 min.^42^ This obviously cannot be applied to continuous monitoring Fe^3+^ ion in real time, especially for monitoring the uptake of metal ions by cells and organisms. Compared to the reported sensor for Fe^3+^ ion, sensor 4 responds rapidly to Fe^3+^ ion within a time (0.5 min) recording on once fluorescence change. Sensor 4 has a low LOD (6.0 × 10^−6^ M) and an appropriate water solubility, as shown in Table S1 in ESI.† In addition, sensor 4 successfully detected Fe^3+^ ion in complex environmental and successfully distinguished it from various metal ions by using 4-treated thin layer plate, showing an excellent anti-interference ability.

## Conclusions

In summary, we developed a novel boronic acid sensor to recognize Fe^3+^ ion reversibly in real time *via* an environmentally friendly method. Sensor 4 responds to Fe^3+^ ion in real time and the blue fluorescence is immediately quenched, which is important for continuous dynamic monitoring of Fe^3+^ ion. In addition, sensor 4 was also used to recognize Fe^3+^ ion successfully in real samples such as rabbit plasma, lake water and tap water. Furthermore, thin layer was processed by sensor 4 to detect Fe^3+^ ion. As a result, it was found that Fe^3+^ ion significantly quenched the fluorescence of thin layer loaded by the sensor 4, while other metal ions were no significant variation under a UV-lamp (365 nm). Therefore, sensor 4 has a great potential to develop into a tool for continuous monitoring and detecting Fe^3+^ ion in real time.

## Conflicts of interest

The authors confirm that this article content has no conflict of interest.

## Supplementary Material

RA-009-C9RA03978C-s001
