# Supplementary material for: A novel boronic acid-based fluorescent sensor for selectively recognizing Fe3+ ion in real time
Source: RSC Adv. 2019 Jun 28;9(35):20306–13. doi: 10.1039/c9ra03978c (PMC9065501; doi:10.1039/c9ra03978c)
Supplement: RA-009-C9RA03978C-s001 [file RA-009-C9RA03978C-s001.pdf]

Electronic Supplementary Material (ESI) for RSC Advances.
This journal is © The Royal Society of Chemistry 2019

Electronic Supplementary Information (ESI)

A novel boronic acid-based fluorescent sensor for selectively recognizing Fe^{3+} ion in real time

Guiqian Fang,[a, b, c, d] Hao Wang,[a, b, c, d] Zhancun Bian,[a, b, c, d] Guo Min,[e] Zhongyu Wu*[a, b, c, d] and Qingqiang Yao*[a, b, c, d]

a. School of Medicine and Life Sciences, University of Jinan-Shandong Academy of Medical Sciences, Jinan 250200, Shandong, China

b. Institute of Materia Medica, Shandong Academy of Medical Sciences, Jinan 250062, Shandong, China

c. Key Laboratory for Biotech-Drugs Ministry of Health, Jinan 250062, Shandong, China

d. Key Laboratory for Rare & Uncommon Diseases of Shandong Province, Jinan 250062, Shandong, China

e. Shandong Province Leather Industrial Research Institute, Jinan 250021, China

*Corresponding authors: E-Mail: wu_med@foxmail.com (Zhongyu Wu), yao_imm@163.com (Qingqiang Yao)

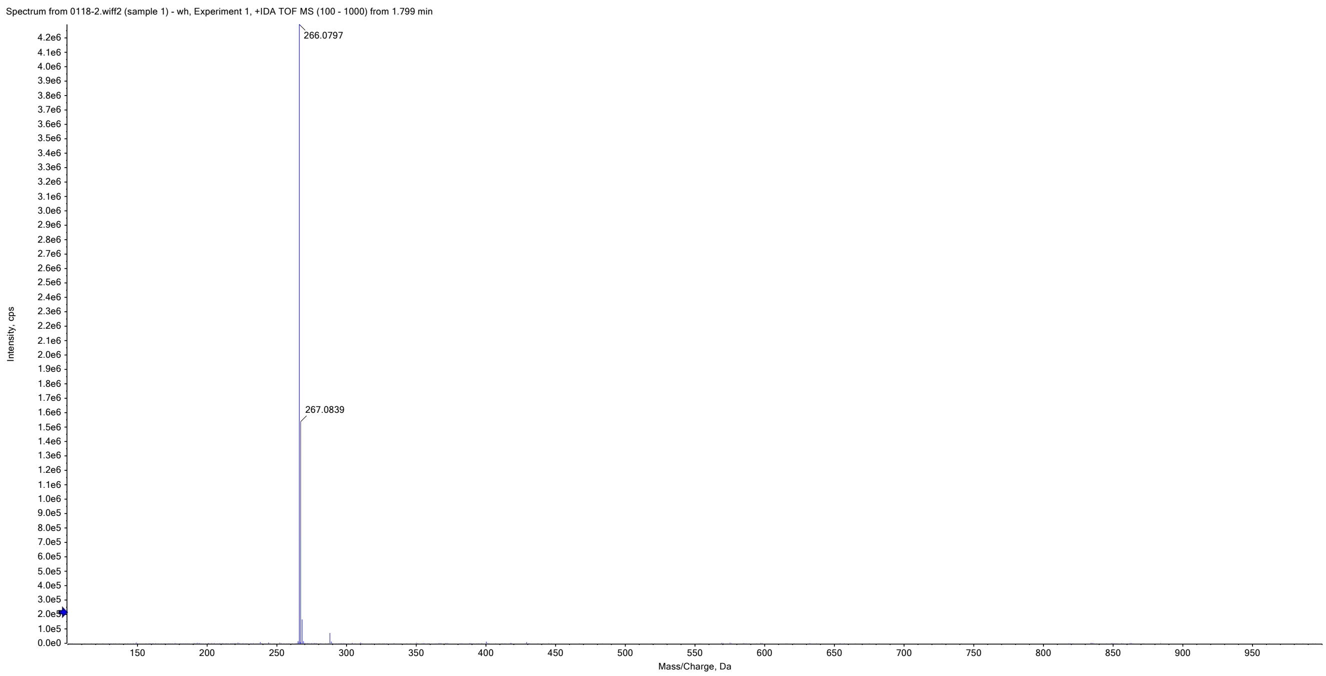

Fig. S1 HRMS spectrum of compound **2**

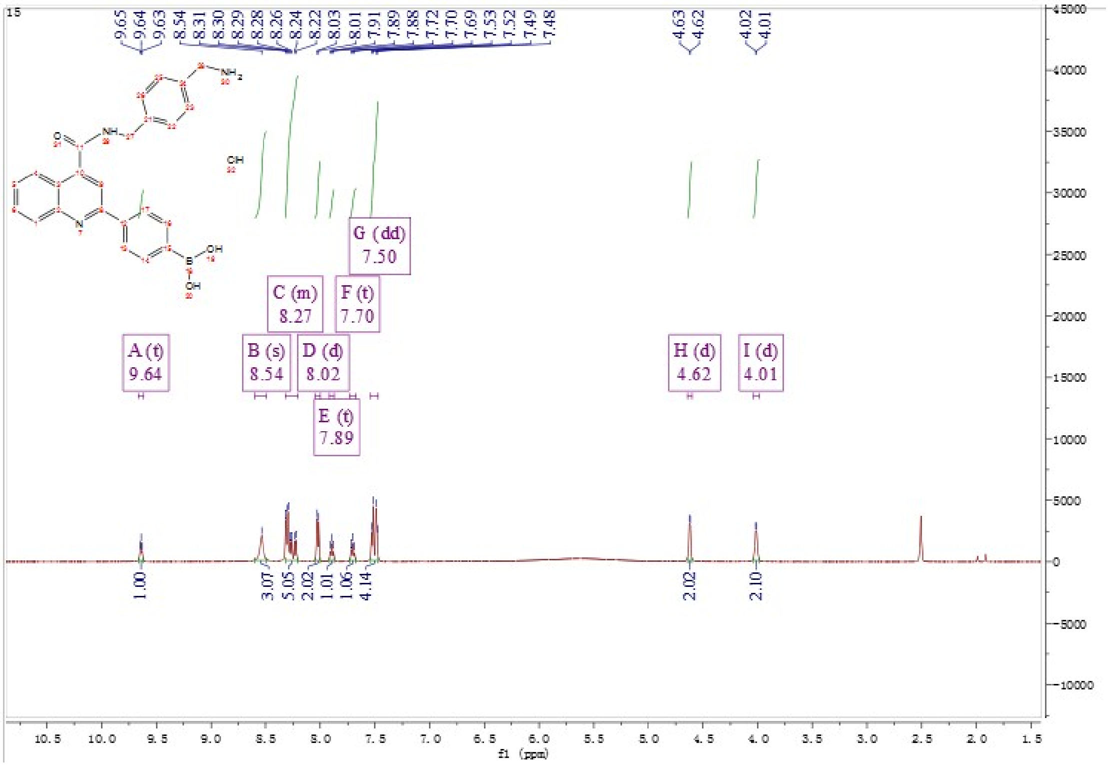

Fig. S2 ^{1}H NMR spectrum of **3**

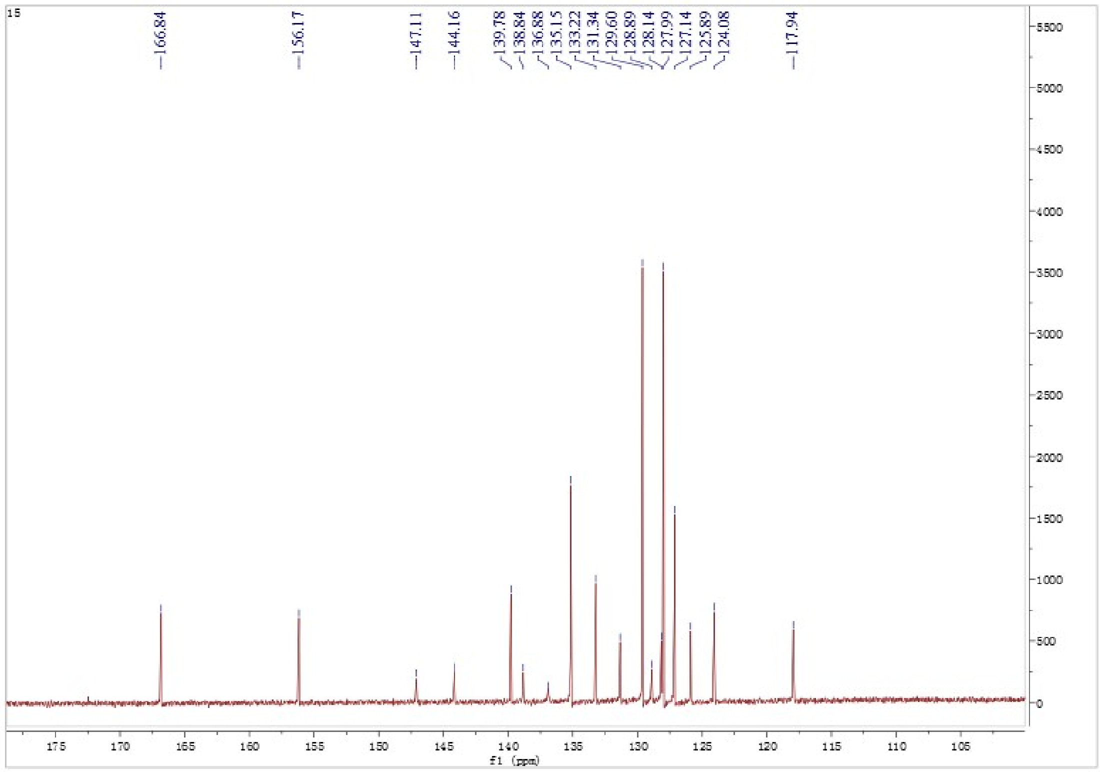

Fig. S3 ^{13}C NMR spectrum of **3**

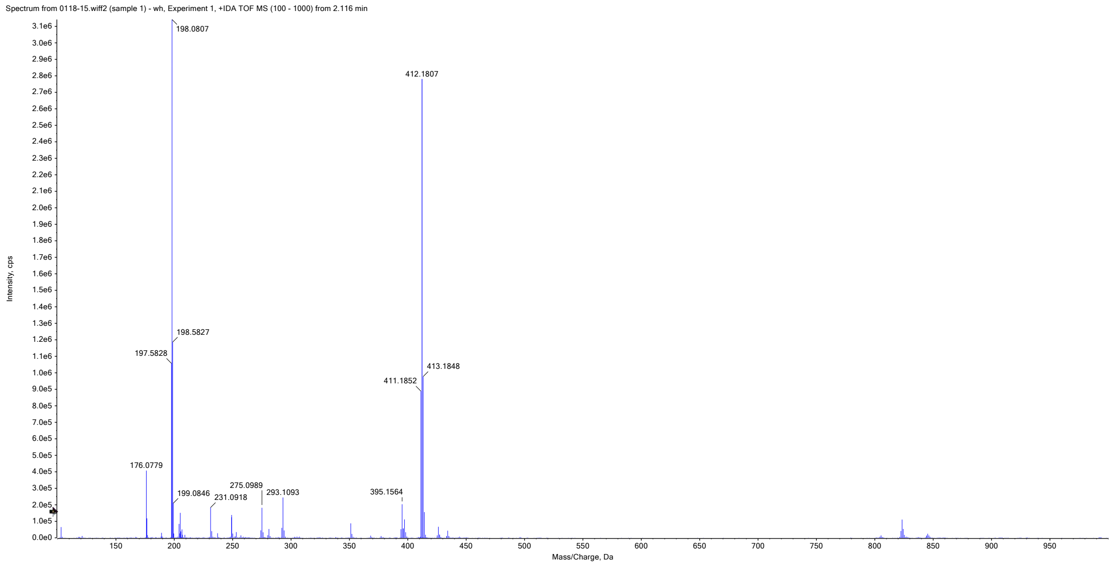

Fig. S4 HRMS spectrum of compound **3**

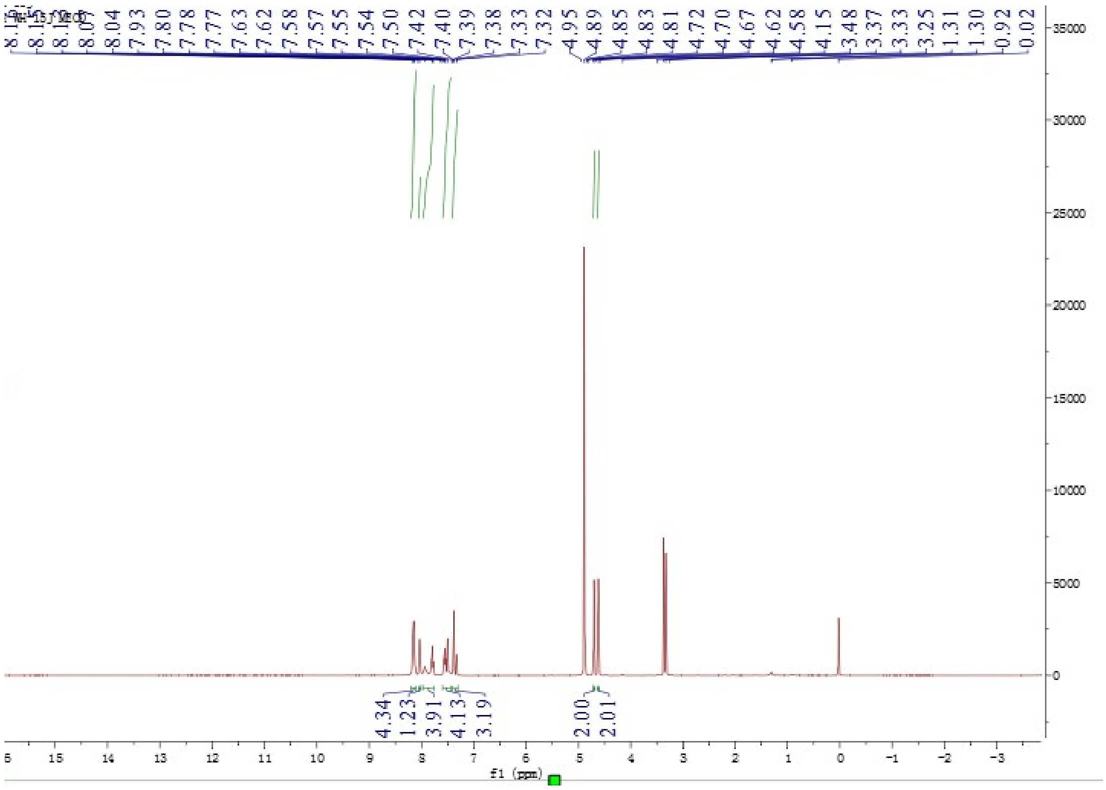

Fig. S5 ^{1}H NMR spectrum of **4**

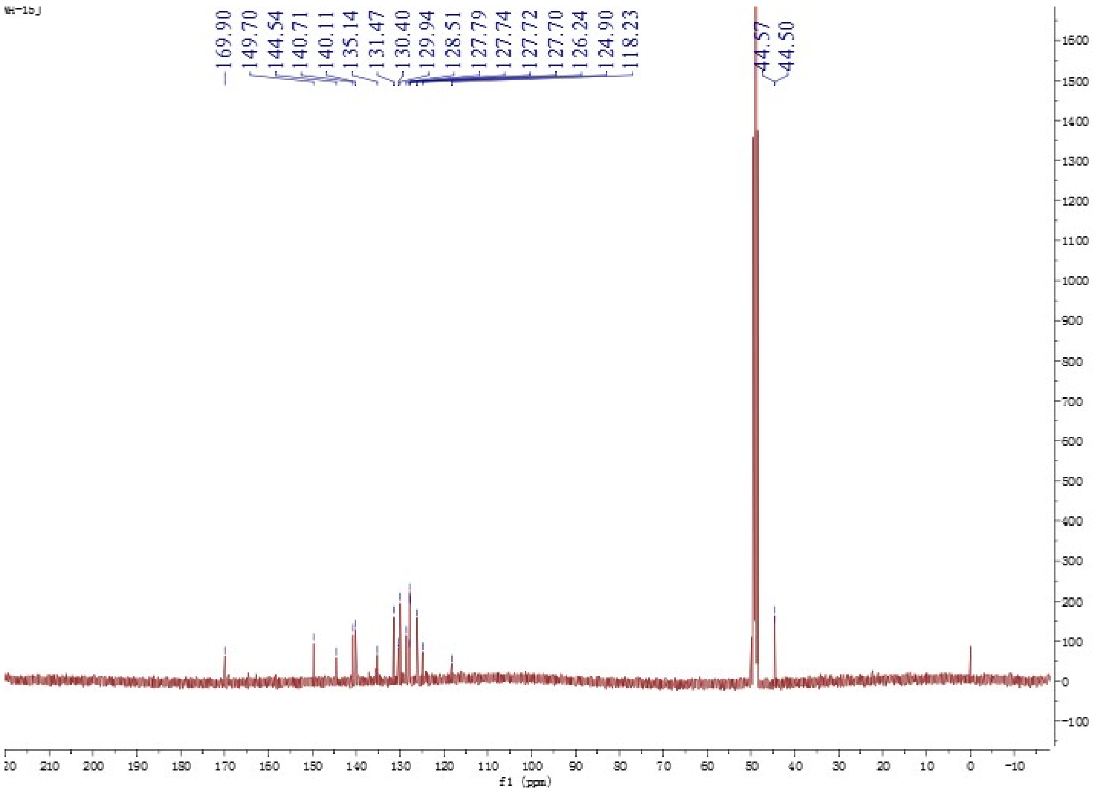

Fig. S6 ^{13}C NMR spectrum of **4**

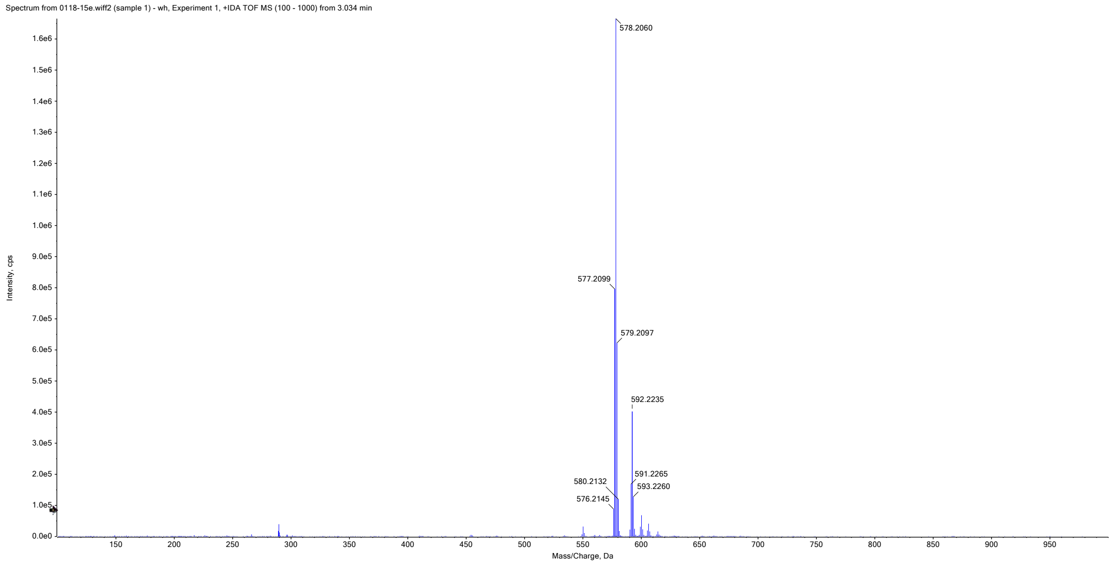

Fig. S7 HRMS spectrum of compound **4**

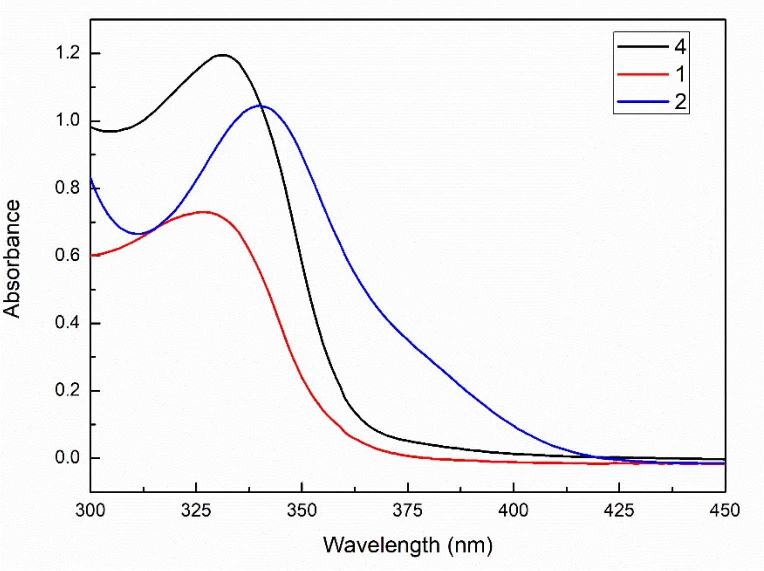

Fig. S8 UV-vis absorption spectra of sensor **1, 2** and **4** in DMSO/H_2O (3:7, v/v).

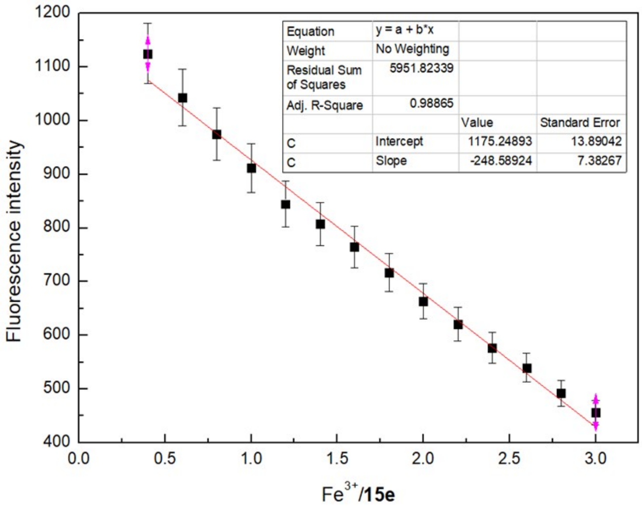

Fig. S9 Linear relationship between sensor **4** and Fe^{3+} ion in DMSO/H_2O (3:7, v/v).

Linear Equation: Y=-248.58924X+1175.24893

R^2 =0.98865

S=2.4858924×10^6

$\delta=\sqrt{\frac{\Sigma(F_i-F_0)^2}{N-1}}$ =5.0 (N=10) K=3

LOD =K × δ/S=6.0 ×10^{-6} M

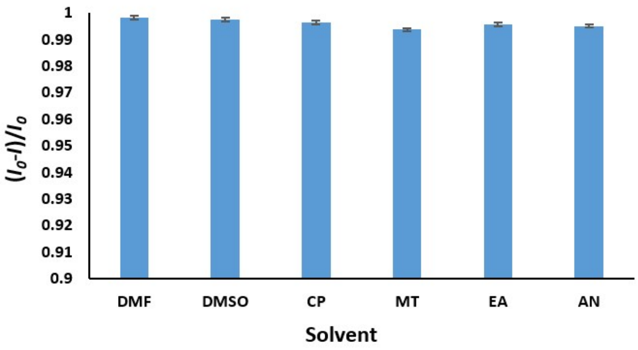

Fig. S10 Relative fluorescence intensity of sensor **4** (1×10^{-4} M) in the presence of 10 equiv. of Fe^{3+} ion in different polar solvents / DMSO (9:1, v/v) solution, at room temperature. From left to right: N, N-dimethylformamide (DMF), dimethylsulfoxide (DMSO), acetone (CP), methyl alcohol (MT), ethyl alcohol (EA), acetonitrile (AN).

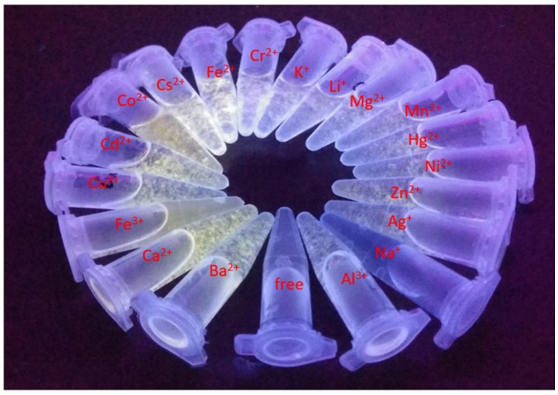

Fig. S11 Photograph of **4** (10^{-4} M) upon adding 10 equiv. of various ions in DMSO/H_2O (1:9, v/v) which was observed under a UV–lamp (365 nm).

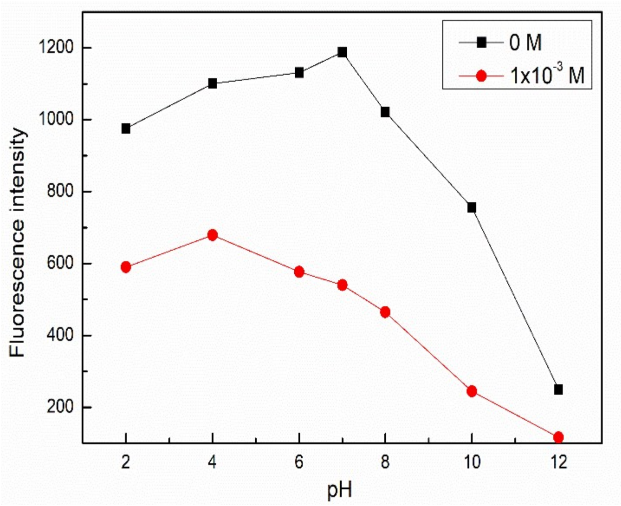

Fig. S12 Fluorescence responses of sensor **4** (1×10^{-4} M) to Fe^{3+} ion in DMSO/H_2O (3:7, v/v, phosphate buffer, 0.1 M) at different pH values. Concentrations of Fe^{3+} ion are given in the plot.

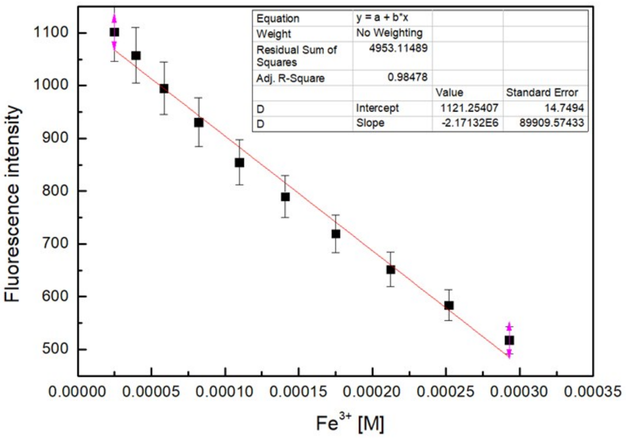

Fig. S13 Linear relationship between sensor **4** and Fe^{3+} ion in rabbit plasma

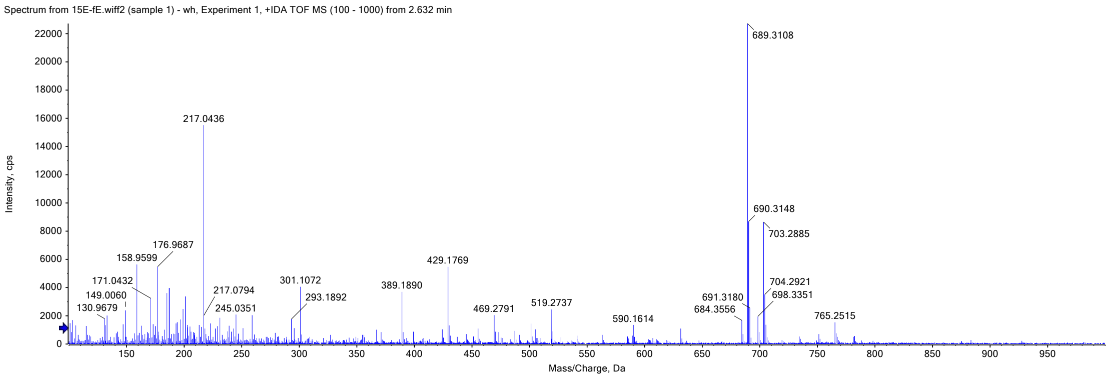

Fig. S14 HRMS spectrum of compound **4**-$2Fe^{3+}$ (calculated 689.0658, found 689.3108).

Table1 Key information of some reported Fe^{3+} sensors

	Sensor	Buffer	Response time	LOD
Gao *et al.* reported[1]	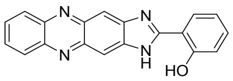	CH_3CN /HEPES(1:4)	60 min	4.8×10^{-6} M
Bao *et al.* reported[2]	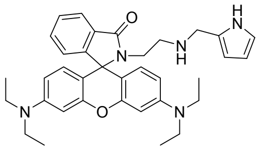	MeOH/H_2O (3:2)	10min	0.031×10^{-6} M

Sepay *et al.* reported[3]	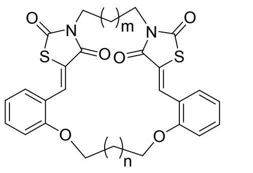	Ethanol/H_2O (3:1)	No data	9.8×10^{-6}M
Nandre *et al.* reported[4]	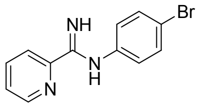	MeOH	No data	0.6×10^{-6} M
Kar *et al.* reported[5]	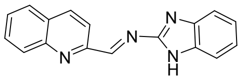	CH_3CN/HEPES(1:4)	1 min	4.0×10^{-6} M
Li *et al.* reported[6]	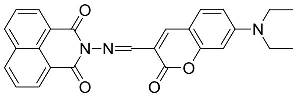	THF/H_2O (1:1)	No data	0.38×10^{-6}M
García-Beltrán *et al.* reported[7]	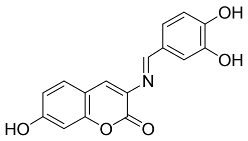	DMSO/HEPES(1:99)	6 min	51.7×10^{-6}M
Dai *et al.* reported[8]	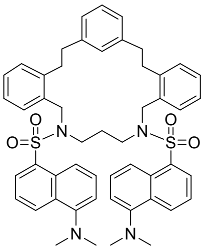	DMSO/HEPES(3:1)	No data	91.1×10^{-6} M
Yao *et al.* reported[9]	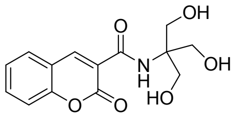	H_2O	No data	No data
Our sensor **4**	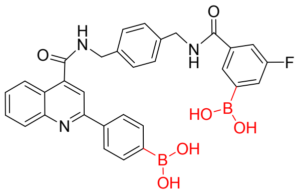	DMSO/ H_2O (3:7)	Within a record time (0.5min)	5.8×10^{-6} M

1. G. Y. Gao, W. J. Qu, B. B. Shi, P. Zhang, Q. Lin, H. Yao, W. L. Yang, Y. M. Zhang and T. B. Wei, *Spectrochimica Acta Part A Molecular & Biomolecular Spectroscopy*, 2014, **121**, 514-519.
2. X. Bao, J. Shi, X. Nie, B. Zhou, X. Wang, L. Zhang, H. Liao and T. Pang, *Bioorganic & Medicinal*

Chemistry, 2014, **22**, 4826-4835.

3. N. Sepay, S. Mallik, P. C. Saha and A. K. Mallik, *New Journal of Chemistry*, 2018, **42**, 15270-15276.
4. J. Nandre, S. Patil, P. Patil, S. Sahoo, C. Redshaw, P. Mahulikar and U. Patil, *Journal of Fluorescence*, 2014, **24**, 1563-1570.
5. C. Kar, S. Samanta, S. Mukherjee, B. K. Datta, A. Ramesh and G. Das, *New Journal of Chemistry*, 2014, **38**, 2660-2669.
6. Z. Li, Y. Zhou, K. Yin, Z. Yu, Y. Li and J. Ren, *Dyes & spigments*, 2014, **105**, 7-11.
7. O. García-Beltrán, B. K. Cassels, C. Pérez, N. Mena, M. T. Núñez, N. P. Martínez, P. Pavez and M. E. Aliaga, *Sensors*, 2014, **14**, 1358-1371.
8. Y. Dai, K. Xu, C. Wang, X. Liu and P. Wang, *Supramolecular Chemistry*, 2017, **29**, 315-322.
9. J. Yao, W. Dou, W. Qin and W. Liu, *Inorganic Chemistry Communications*, 2009, **12**, 116-118.
